# Comprehensive Evaluation of Coal-Fired Bottom Slag as a Precursor for Alkali-Activated Materials

**DOI:** 10.3390/ma19183896

**Published:** 2026-09-13

**Authors:** Jie Wen, Yana Mao, Yongfeng Wei

**Affiliations:** 1 Gansu Academy of Sciences, Lanzhou 730000, China; wenjie129@sohu.com; 2 Gansu Natural Energy Research Institute, Lanzhou 730000, China

**Keywords:** bottom slag, alkali-activated materials, compressive strength, reaction mechanism, microstructure

## Abstract

Bottom slag (BS), a coal combustion by-product, is an underutilized aluminosilicate resource with potential for alkali activation. In this study, the physicochemical characteristics of BS, including its chemical composition, mineralogy, morphology, and activity index, were systematically evaluated, and ground BS was blended with ground granulated blast-furnace slag (GGBFS) to prepare alkali-activated binders. The effects of BS content on mechanical properties, reaction products, and microstructural evolution were investigated using compressive strength tests and multi-scale characterization techniques. The results showed that BS contains abundant amorphous aluminosilicate phases with low residual carbon and exhibits considerable latent cementitious activity despite its relatively low intrinsic reactivity. The mechanical performance of the binders was strongly governed by precursor composition. Incorporating 20–40 wt.% BS achieved the optimum balance between precursor reactivity and strength development, whereas higher BS contents significantly inhibited alkali activation reaction because of the dilution of reactive glassy phases. Strength development was closely associated with the formation of C-(A)-S-H and N-A-S-H gels and the development of a dense microstructure. These results demonstrate that the synergistic activation of BS and GGBFS provides an effective route for producing low-carbon alkali-activated binders while enabling the high-value utilization of coal-fired BS.

## 1. Introduction

Coal remains the world’s most abundant fossil energy resource, with proven reserves of approximately 1.14 trillion tons and accounting for over half of the current global primary energy supply [1,2]. Although renewable energy has expanded rapidly in recent decades, coal continues to dominate electricity generation, steel production, district heating, and numerous energy-intensive industries. Globally, coal-fired power plants consume approximately 5–6 billion tons of coal each year, generating an estimated 0.7–1.0 billion tons of coal combustion solid wastes. Fly ash has been widely valorized in cement and concrete production owing to its favorable physicochemical properties, and in some regions its supply has become insufficient to satisfy industrial demand. By contrast, BS, representing approximately 20–30% of total coal combustion residues, remains largely underutilized and is commonly stockpiled or landfilled, resulting in substantial environmental burdens and inefficient resource utilization.

BS is the coarse residue discharged from the bottom of coal-fired boilers after the high-temperature melting of inorganic mineral constituents during combustion. The mineralogical composition and reactivity of BS are closely associated with furnace temperatures, which generally range between 1300 and 1700 °C. Because of its irregular morphology, porous texture, and relatively low intrinsic activity, untreated BS cannot be directly employed as a cementitious material [3,4,5]. Consequently, extensive efforts have focused on mechanically processing BS into fine particles for application as aggregate replacement or supplementary cementitious material in concrete. Previous investigations have demonstrated that ground BS can improve mortar workability through internal water release and the ball-bearing effect of spherical particles, while its major chemical constituents (SiO_2_, Al_2_O_3_, and Fe_2_O_3_) and mineral phases (quartz, mullite, feldspar, and magnetite) indicate considerable potential for pozzolanic or alkali-activated reactions [6,7,8]. Although incorporating BS generally decreases early-age strength, long-term strength development can be significantly enhanced owing to secondary hydration and pozzolanic reactions [9,10]. Moreover, the porous structure of BS provides excellent thermal insulation and acoustic absorption, enabling its application in lightweight construction materials.

Baite et al. [11] investigated the volumetric replacement of fine aggregate (10–100%) with bottom slag possessing a specific gravity of 2220 kg/m^3^ and an apparent density of 770 kg/m^3^. As the replacement ratio increased from 0 to 50%, the mortar consistency increased markedly from approximately 17 mm to 35 mm. However, further increasing the bottom slag content resulted in a gradual decline in consistency. Kim et al. [12] incorporated ground bottom slag as a supplementary cementitious material in cement mortar and observed that, despite the higher initial water absorption of the fine particles, the stored water was subsequently released during mixing, thereby enhancing mortar workability by approximately 10–20%. In agreement with these findings, Bai et al. [13] attributed the improved workability to the “ball-bearing effect” of the spherical particles present in ground bottom slag, which effectively reduced the water requirement of concrete.

According to the studies of Shi Jianxin and Cheriaf M et al. [14], the chemical composition of bottom slag is dominated by SiO_2_, Al_2_O_3_, and Fe_2_O_3_, whereas its principal crystalline phases consist of quartz, mullite, feldspar, and magnetite. Kula et al. [15] reported that replacing composite Portland cement with ground bottom slag reduced the early-age strength of cementitious composites, and the strength reduction became more pronounced with increasing replacement level. Nevertheless, all mixtures achieved higher compressive strength than the control at 28 days, indicating that the pozzolanic reaction of bottom slag contributed significantly to the later-age strength development.

Apart from its application as a cementitious constituent, the lightweight and highly porous nature of bottom slag has also promoted its use in thermal and acoustic insulation materials. Hannan et al. [16] demonstrated that concrete panels incorporating a high volumetric replacement level (≥80%) of bottom slag in place of fine aggregate were capable of absorbing more than 30% of incident sound. Moreover, studies by Zhang and Torkittikul et al. [17,18] showed that replacing crushed stone with bottom slag significantly enhanced the thermal insulation performance of concrete, as evidenced by the continuous reduction in thermal conductivity with increasing bottom slag content.

Despite these advantages, the large-scale valorization of BS remains limited because its latent reactivity has not been sufficiently activated. Existing studies have mainly focused on the utilization of BS as aggregate replacement, supplementary cementitious material, or thermal and acoustic insulation material. In these applications, BS is generally considered a low-reactivity material, while its potential as an alkali-activated precursor has received comparatively limited attention. More importantly, owing to its relatively low intrinsic reactivity, the direct utilization of BS as a primary precursor remains challenging. Although blending low-reactivity industrial solid wastes with highly reactive ground granulated blast-furnace slag (GGBFS) may provide an effective approach, the synergistic effect of BS and GGBFS on the reaction process and performance development of alkali-activated materials has not been sufficiently clarified. Previous studies have also mainly focused on the macroscopic properties of BS-containing cementitious materials, whereas systematic investigations into the relationships among precursor composition, reaction products, microstructural evolution, and mechanical performance remain limited. Therefore, further research is required to clarify the activation mechanism of BS and establish an effective strategy for its high-value utilization in alkali-activated materials.

Accordingly, this study systematically characterizes the physicochemical properties of BS, including its chemical composition, mineralogy, morphology, and activity index, to assess its suitability as an alkali-activated precursor. Ground BS was then blended with GGBFS to synthesize BS-based alkali-activated materials (BS-based AAMs). The effects of precursor composition on mechanical properties, phase evolution, and microstructural development were comprehensively investigated using mechanical testing and multi-scale characterization techniques. The underlying reaction mechanisms governing the formation of BS-based AAMs were elucidated, providing theoretical guidance and a feasible technical route for the resource-efficient, large-scale, and high-value utilization of BS in sustainable construction applications.

## 2. Materials and Methods

### 2.1. Raw Materials

#### 2.1.1. Bottom Slag

**Figure 1:**
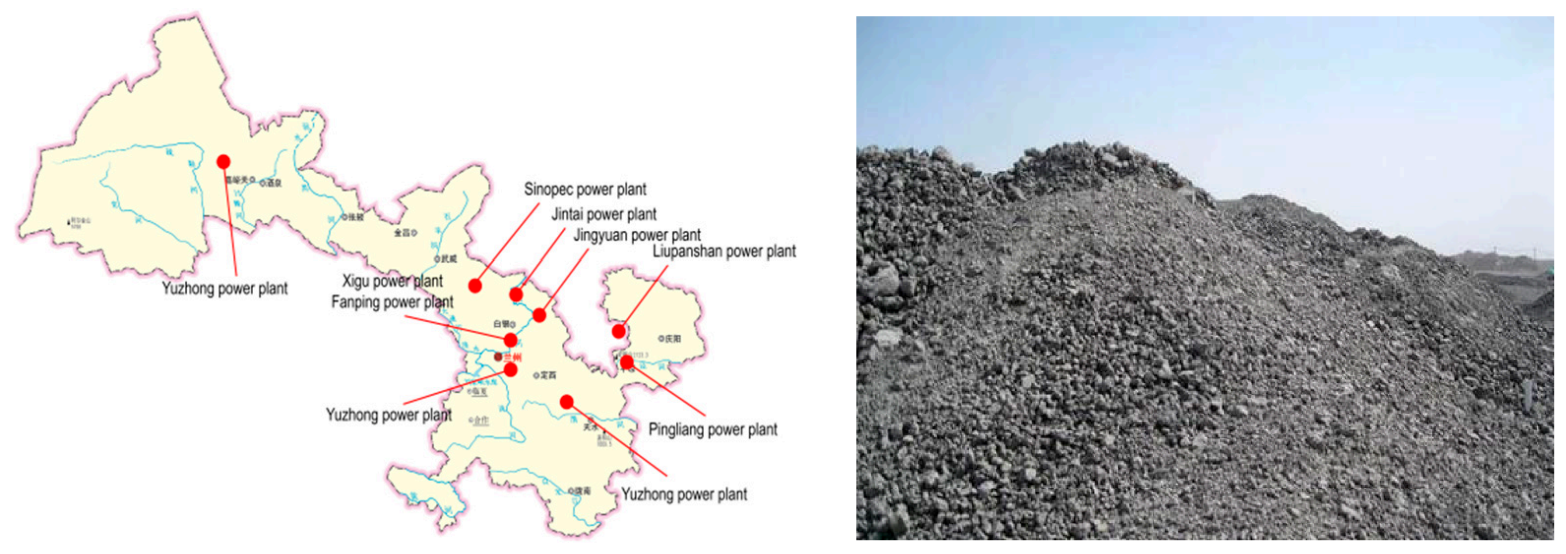
BS sampling and disposal sites.

The BS used in this study was collected from clinker-like solid residues discharged from the bottom of coal-fired power plant boilers. It is typically characterized by a grayish-black color, a porous structure, and irregular granular or lumpy morphology. Compared with natural aggregates, BS possesses lower compressive strength, higher porosity, and lower density than natural sand and gravel (Figure 1). The BS samples were obtained from ten coal-fired power plants located in different regions of Gansu Province, China.

#### 2.1.2. Ground Granulated Blast Furnace Slag

The GGBFS used in this study was sourced from the Jiuquan Iron and Steel Company (Jiayuguan, China). The chemical composition of the raw materials is presented in Table 1.

#### 2.1.3. Alkali Activator

The alkali activators were created using pre-mixed reagent-grade sodium silicate powder with 22.1 wt.% SiO_2_, 22.8 wt.% Na_2_O, and 55.1 wt.% H_2_O, reagent-grade sodium hydroxide pellets with 99 wt.% purity and distilled water. Different modulus alkali activators were prepared and categorized in Table 2. The reagent-grade sodium silicate powder was provided by Qingdao Haiwan Chemical Group Co., Ltd. (Qingdao, China), and the reagent-grade sodium hydroxide pellets were provided by Tianjin Chemical Reagent Company (Tianjin, China).

### 2.2. Sample Preparation

#### 2.2.1. BS Powder

The BS powder is a solid powder obtained by drying, crushing, and grinding bottom slag collected from a power plant. All BS powder was ground to a fineness with a 45 μm sieve residue of 1.5–2.5%. BS powder was primarily used for the testing of raw BS materials, including loss on ignition (LOI), XRF, XRD, and SEM, as well as for the preparation of paste and mortar samples.

#### 2.2.2. BS-Based Composite Mortar

The BS-based composite mortar was used to evaluate the pozzolanic reactivity of BS; it was a BS-based composite mortar containing 30 wt.% BS powder as a cement replacement. The BS-based composite mortar was prepared by mixing BS powder, P.O 42.5 Portland cement, water, and ISO standard sand at a mass ratio of 135:315:225:1325 using a JJ-5 mortar mixer supplied by Wuxi Jianding Instrument Company (Wuxi, China). The preparation of test specimens followed the procedures specified in Standard GB/T 1596(CN) [19]. The BS powder used in this batch of samples was obtained from the Yuzhong Power Plant (Lanzhou, China).

#### 2.2.3. BS-Based AAM Paste

The BS-based AAM paste samples were created by mixing BS powder, GGBFS, alkali activator, and water using a mortar mixer. The material ratios for each group of samples are listed in Table 3. All paste samples were shaped into 20 mm × 20 mm × 20 mm blocks using steel cube molds and left to cure in the mold for 24 h. In total, 12 parallel specimens were prepared for each BS-based AAM paste. After this initial curing period, the blocks were removed from the mold and transferred to a standard curing chamber, kept at 20 °C and a relative humidity of 95%. The blocks were then used for XRD and SEM analyses. The BS powder used in this batch of samples was obtained from the Yuzhong Power Plant (Lanzhou, China).

#### 2.2.4. BS-Based AAM Mortar

BS-based AAM mortar samples were made utilizing BS powder, GGBFS, alkali activator, standard sand, and water. The ratios of the materials used are presented in Table 4. Each mortar sample produced a batch of blocks with dimensions of 40 mm × 40 mm × 160 mm; the preparation of test specimens followed the procedures specified in Standard GB/T 29417(CN) [20]. 18 parallel specimens were prepared for each BS-based AAM mortar. The mortar blocks were cured in the same environmental chamber as the paste samples, and those paste samples were utilized for the strength tests. The BS powder used in this batch of samples was obtained from the Yuzhong Power Plant (Lanzhou, China).

### 2.3. Experimental Program

#### 2.3.1. Characterization

To determine the chemical composition of the raw materials, a Rigaku X-ray fluorescence spectrometer (XRF) manufactured by Rigaku Corporation (Tokyo, Japan) was utilized. The mineral crystal structure was identified using an Ultima IV X-ray diffractometer (XRD) manufactured by Rigaku Corporation (Tokyo, Japan)., with a Cu Kα source and an excitation voltage of 40 kV at 30 mA. The microstructure and elemental distribution were analyzed using a Thermo Scientific Apreo S scanning electron microscope (SEM) manufactured by Thermo Fisher Scientific (Waltham, MA, USA). The density was measured according to the GB/T 208 (CN) standard [21], with anhydrous kerosene used as the medium. The loss on ignition was determined following the GB/T 176(CN) standard [22], with the calcination temperature set at 950 ± 25 °C.

#### 2.3.2. Mechanical Test

All mortar specimens were cured at 20 ± 2 °C and 95% relative humidity (RH). Compressive strength tests were conducted at curing ages of 7, 28, and 56 days. For each testing age, three parallel specimens were tested, and the arithmetic mean of the three individual measurements was calculated and reported as the compressive strength for that specific curing age. The compressive strength of the reference OPC mortar is presented in Table 5. The mechanical strength test of the mortar was determined in accordance with the Standard GB/T 17671(CN) [23].

## 3. Results and Discussion

### 3.1. BS Material

#### 3.1.1. Morphological Characteristics

**Figure 2:**
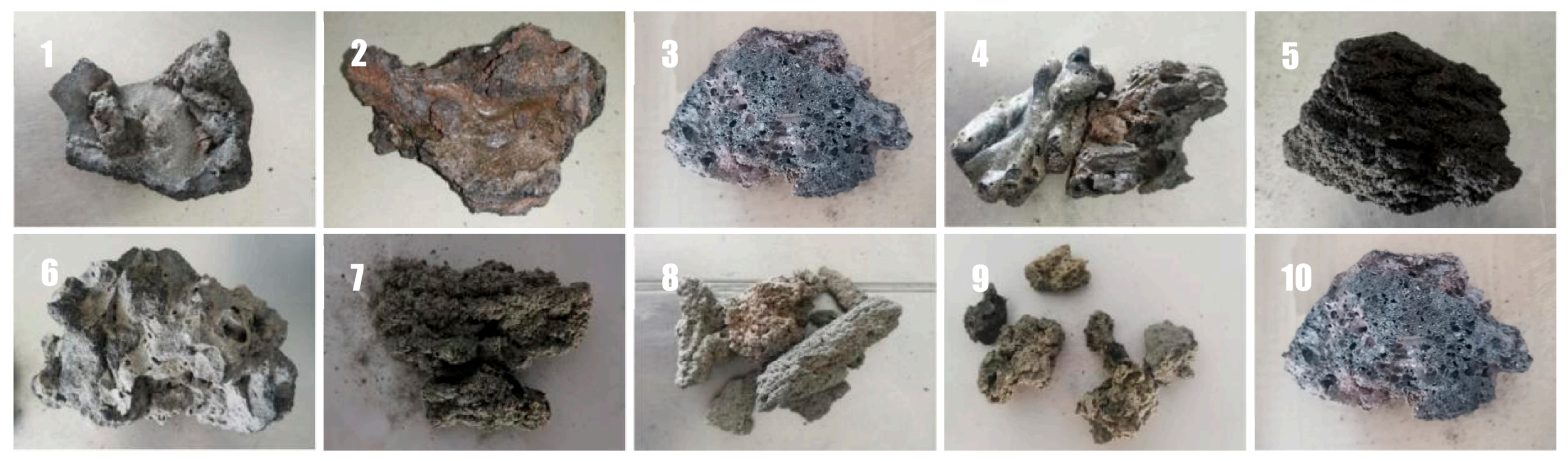
Morphological characteristics of BS from different power plants. (**1**) Jingtai plant, (**2**) Jingyuan plant, (**3**) Yuzhong plant, (**4**) Fanping plant, (**5**) Xigu plant, (**6**) Pingliang plant, (**7**) Yuanyanghu plant, (**8**) Sinopec plant, (**9**) Liupanshan plant, (**10**) Changle plant.

As shown in Figure 2, the BS samples collected from different power plants exhibit porous, sintered mineral structures with smooth surfaces, high hardness, and abundant glassy phases, while no residual carbonaceous organic matter is observed. Significant differences in the macroscopic morphology are evident among the BS samples from different power plants. Some samples exhibit a high degree of sintering and vitrification, whereas others show relatively poor sintering and vitrification. In the latter case, the blocky particles are formed by the agglomeration of finer particles rather than by complete melting and solidification. By comparing the boiler technologies employed at different power plants, these morphological differences are considered to be closely associated with variations in BS discharge temperature and boiler operating processes.

#### 3.1.2. LOI Characteristics

**Figure 3:**
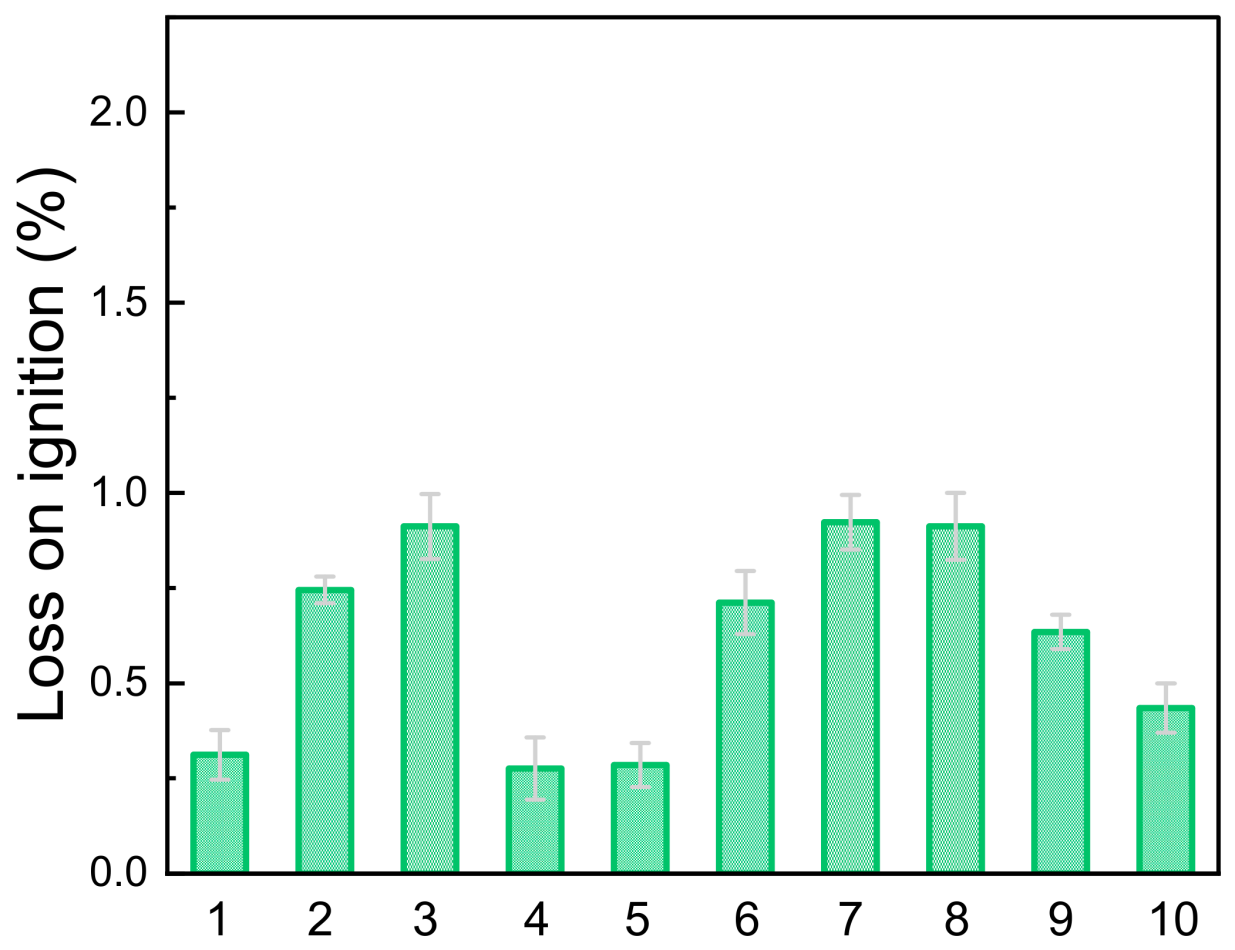
LOI of BS powder from different power plants. (1) Jingtai plant, (2) Jingyuan plant, (3) Yuzhong plant, (4) Fanping plant, (5) Xigu plant, (6) Pingliang plant, (7) Yuanyanghu plant, (8) Sinopec plant, (9) Liupanshan plant, (10) Changle plant.

LOI, which reflects the amount of gaseous products (e.g., H_2_O and CO_2_) released during thermal decomposition, as well as the organic matter content of a material, is a key parameter for evaluating the stability and chemical reactivity of different BS solid wastes. As shown in Figure 3, the LOI values of all BS samples are highly consistent and remain below 1.0%, which is considerably lower than the maximum allowable limit of 10% specified for low-calcium fly ash. These results indicate that the BS produced by different power plants across Gansu Province contains only a small amount of organic matter and exhibits good compositional uniformity.

#### 3.1.3. Microstructural Characteristics

**Figure 4:**
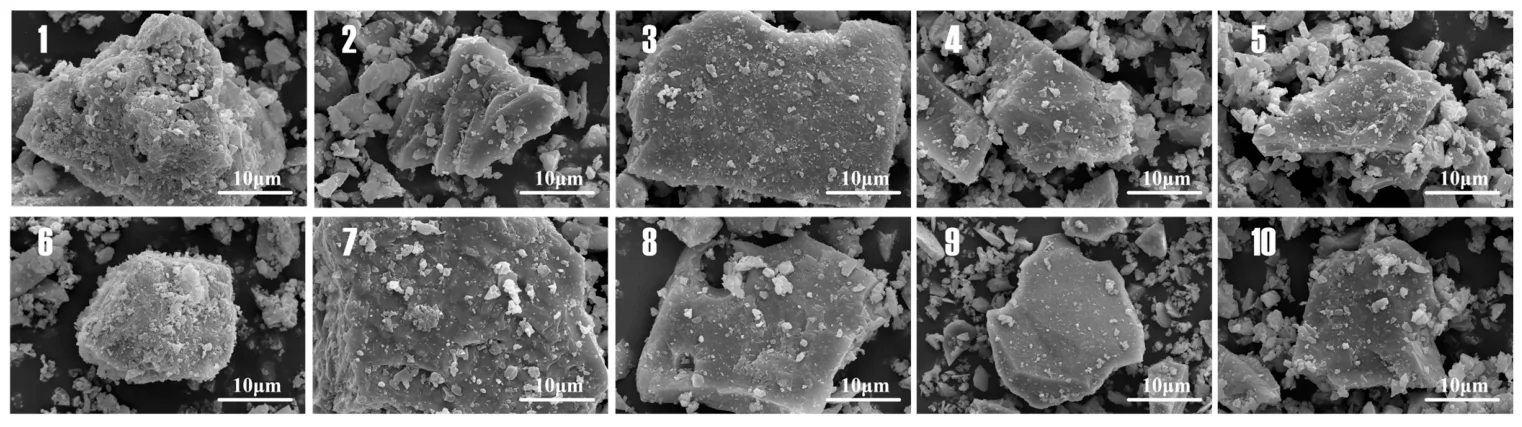
SEM of BS from different power plants. (**1**) Jingtai plant, (**2**) Jingyuan plant, (**3**) Yuzhong plant, (**4**) Fanping plant, (**5**) Xigu plant, (**6**) Pingliang plant, (**7**) Yuanyanghu plant, (**8**) Sinopec plant, (**9**) Liupanshan plant, (**10**) Changle plant.

As shown in Figure 4, the microstructures of the different ground BS materials are distinctly different from the spherical glassy particles of fly ash. After crushing and grinding, all BS samples exhibit irregular fractured morphologies with dense structures and only a small number of pores, resembling the morphology of finely ground cement clinker. Compared with the spherical glassy particles of fly ash, these irregular fractured particles possess higher chemical reactivity, which facilitates the leaching of active components and promotes subsequent pozzolanic reactions.

#### 3.1.4. Chemical Composition

The X-ray fluorescence (XRF) analysis results presented in Table 6 show that all BS samples exhibit similar chemical compositions. The predominant oxides are SiO_2_, Al_2_O_3_, Fe_2_O_3_, and CaO, with their combined content exceeding 95 wt.%, indicating that these four oxides constitute the principal mineral components of the BS.

The chemical composition of BS was dominated by SiO_2_, accounting for approximately 55–65 wt.%, followed by Al_2_O_3_ at around 20 wt.%. Fe_2_O_3_ ranked third with a content of approximately 6–10 wt.%, while CaO exhibited the lowest proportion, accounting for about 6 wt.%. The chemical composition indicates that BS collected from different power plants is consistently characterized as a typical aluminosilicate material. Moreover, the chemical compositions of BS from different regions showed good stability with only minor variations. Owing to its similarity to conventional pozzolanic materials in terms of chemical composition, BS possesses the fundamental material basis for the production of pozzolanic binders and geopolymer cementitious materials.

#### 3.1.5. Mineral Composition

**Figure 5:**
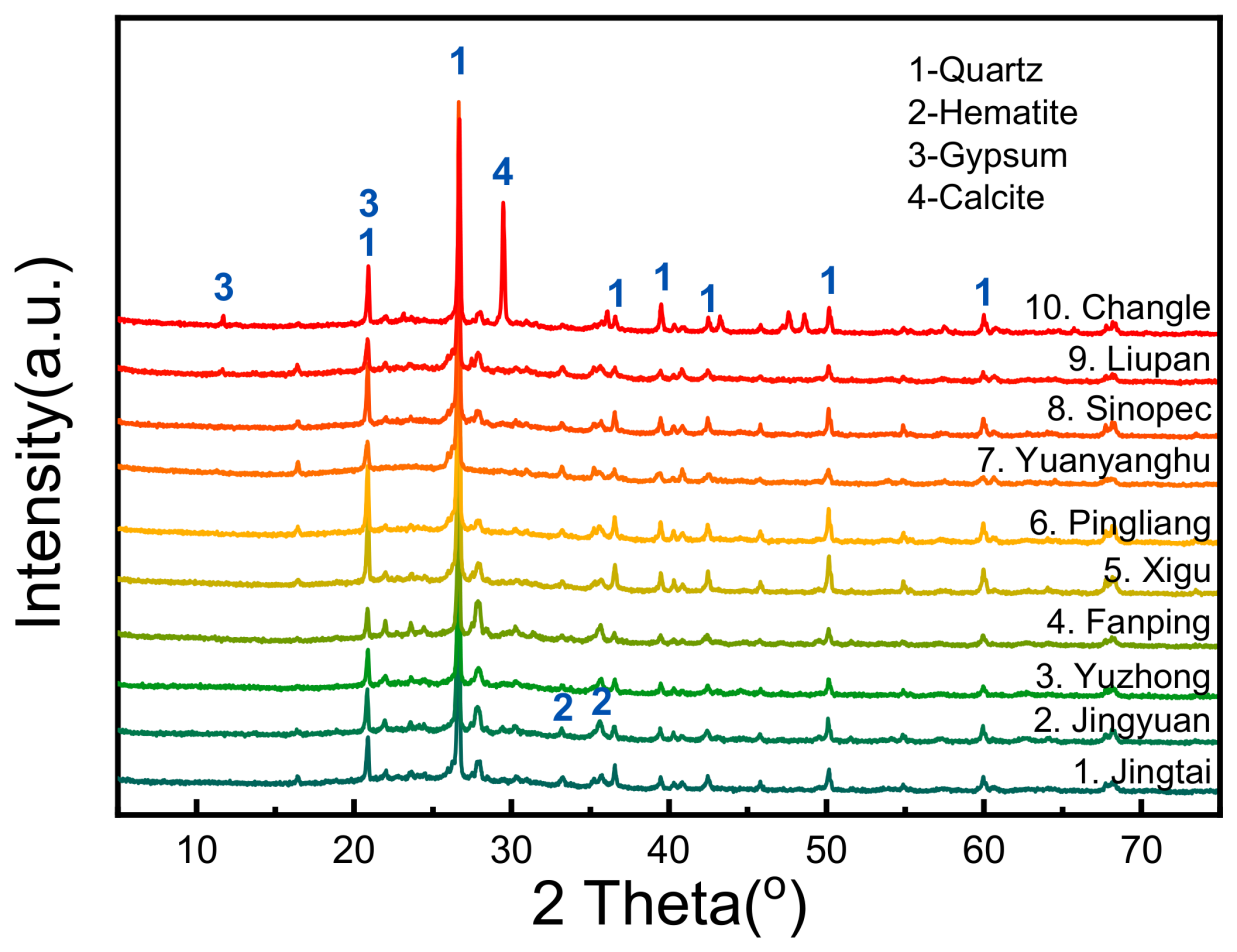
XRD patterns of BS from different power plants.

As illustrated in Figure 5, the XRD results reveal that the crystalline mineralogy of the BS samples is relatively uncomplicated, with quartz being the dominant crystalline phase accompanied by a small quantity of hematite. Diffraction peaks of gypsum and calcite were detected only in the BS from the Changle Power Plant. In addition, a distinct amorphous hump was observed in all samples over the 2θ range of 25–30°, indicating the presence of glassy aluminosilicate phases generated during high-temperature melting. These amorphous aluminosilicate phases are generally recognized as the principal contributors to the cementitious reactivity of the BS.

#### 3.1.6. Strength Properties

**Figure 6:**
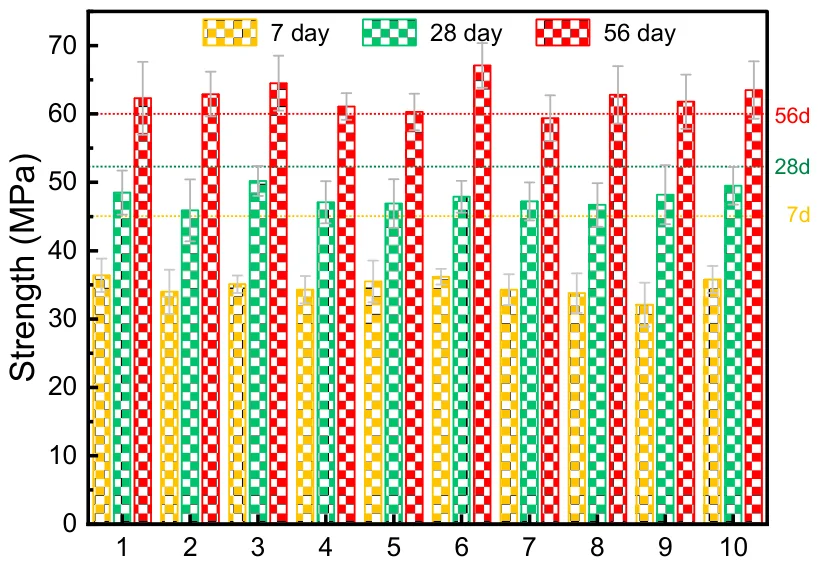
Activity index (compressive strength) of BS from different power plants. (1) Jingtai plant, (2) Jingyuan plant, (3) Yuzhong plant, (4) Fanping plant, (5) Xigu plant, (6) Pingliang plant, (7) Yuanyanghu plant, (8) Sinopec plant, (9) Liupanshan plant, (10) Changle plant.

To evaluate the chemical reactivity of BS, 30 wt.% of the cement was replaced with ground BS, and the strength activity index was adopted as the evaluation criterion. The test results shown in Figure 6 indicate that the incorporation of ground BS considerably reduced the early compressive strength of mortar. At a curing age of 3 days, the compressive strength of BS-containing mortars was only 70–80% of that of the reference mortar. However, a pronounced increase in strength was observed with prolonged curing. At 28 days, the compressive strength exceeded 90% of the reference value, and after 56 days, almost all BS-blended mortars achieved strengths higher than the control. Among them, the mortar prepared with 6# Pingliang BS showed the highest strength, approximately 12% greater than that of the reference mortar.

The inferior early-age strength is primarily associated with the limited pozzolanic activity of BS resulting from its high degree of crystallinity and relatively low glass-phase content. In addition, replacing cement with BS introduces a dilution effect that decreases the quantity of hydration products formed at early ages. As curing continues, Ca(OH)_2_ generated from cement hydration gradually activates the reactive silica and alumina phases in BS, leading to the formation of additional C-A-S-H gel through secondary hydration reactions and refinement of the pore structure. Consequently, the compressive strength increased to approximately 95% of the reference value at 28 days and eventually exceeded the reference by about 10% at 56 days, owing to the continuous densification of the matrix and enhancement of the interfacial transition zone.

#### 3.1.7. Formation Mechanism

**Figure 7:**
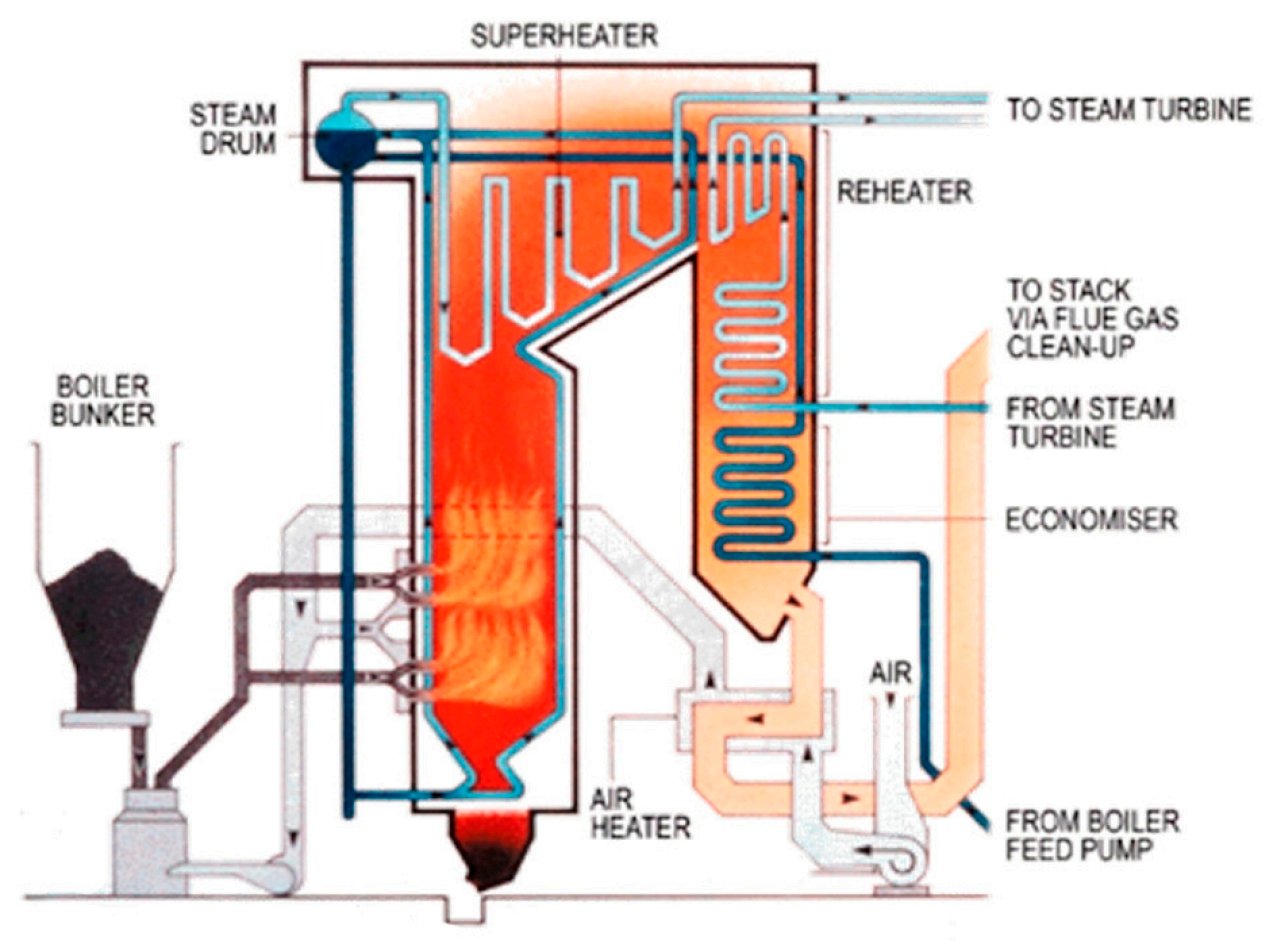
Schematic illustration of a pulverized coal-fired boiler.

BS is an irregular clinker-like by-product generated at the bottom of pulverized coal-fired boilers during coal combustion (as shown in Figure 7). Since the flame temperature in pulverized coal boilers typically ranges from 1300 °C to 1700 °C, exceeding the thermal transformation temperatures of kaolinite-derived phases, BS contains abundant molten aluminosilicate glass formed during the combustion process. This amorphous glass phase is structurally similar to the reactive vitreous phase found in cementitious materials. Owing to its long-range disordered atomic arrangement, the aluminosilicate glass possesses relatively low chemical stability and consequently exhibits high chemical reactivity.

XRF analysis indicates that BS is primarily composed of SiO_2_, Al_2_O_3_, Fe_2_O_3_, CaO, and MgO. The inorganic fraction of coal mainly consists of quartz and kaolinite. Quartz, a highly stable trigonal crystalline oxide, retains its crystal structure during combustion because its Si–O bonds are resistant to thermal rupture. Consequently, quartz contributes little to the chemical activity of BS.

In contrast, the reactivity of BS is predominantly associated with the thermal transformation of kaolinite. Kaolinite is a 1:1 layered aluminosilicate mineral constructed from alternating [SiO_4_] tetrahedral and [AlO_6_] octahedral sheets. Although the layered structure is thermodynamically stable under ambient conditions, thermal treatment induces progressive structural evolution. Below 400 °C, kaolinite experiences negligible structural alteration. Between approximately 400 and 950 °C, particularly within the optimal activation range of 600–900 °C reported in previous studies, dehydroxylation destroys the ordered layered structure and generates poorly crystalline metakaolinite. This metastable phase consists primarily of Si–O and Al–O structural units in the form of [SiO_4_] tetrahedra together with [AlO_4_] tetrahedra or [AlO_6_] octahedra. These disordered structural units are susceptible to dissolution under alkaline conditions, followed by polycondensation reactions that produce geopolymeric gels with zeolitic frameworks and other hydration products. When the calcination temperature exceeds about 950 °C, metakaolinite undergoes recrystallization to form aluminosilicate spinel and mullite. The development of these stable crystalline phases substantially decreases the chemical activity of the material, making it unsuitable as a reactive cementitious precursor.

### 3.2. BS-Based Alkali-Activated Material

#### 3.2.1. Strength

**Figure 8:**
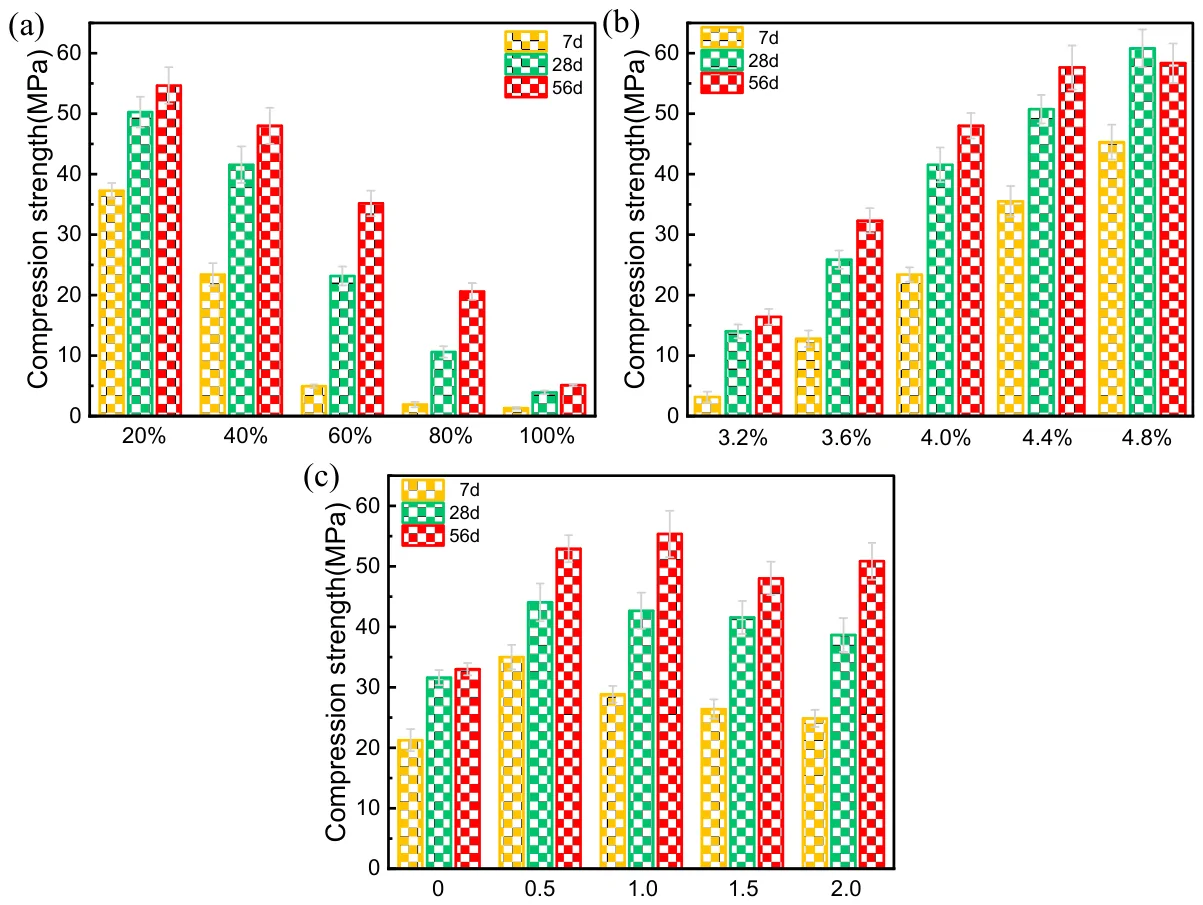
Compressive strength of BS-based AAMs. (**a**) Varying BS content. (**b**) Varying alkali activator dosages. (**c**) Varying alkali activator moduli.

Figure 8a depicts the compressive strength evolution of alkali-activated BS/GGBFS binders containing different proportions of BS. Increasing the BS replacement level resulted in a continuous reduction in compressive strength, with the deterioration being considerably more pronounced at early curing ages than at later ages. The optimum performance was achieved at a BS content of 20%, yielding compressive strengths of 37.25, 50.25, and 54.65 MPa after 7, 28, and 56 days of curing, respectively. Increasing the BS content to 40% reduced the corresponding strengths to 23.40, 41.55, and 48.00 MPa, whereas a further increase to 80% led to a substantial decline to only 1.89, 10.60, and 20.60 MPa, respectively. When BS completely replaced GGBFS as the precursor, the binder developed negligible mechanical strength. These findings demonstrate that BS can serve as a precursor for alkali-activated binders only when blended with GGBFS, owing to its substantially lower chemical reactivity compared with GGBFS.

The effect of activator dosage on strength development is presented in Figure 8b. The compressive strength increased progressively with increasing activator dosage, confirming that the dosage of the alkaline activator is a critical factor controlling the geopolymerization process. Nevertheless, the rate-of-strength gain gradually diminished once the dosage exceeded 1.1, suggesting that excessive activator addition provides limited further improvement. Moreover, increasing the activator dosage had a more significant influence on early-age strength than on long-term strength. At an activator dosage of 0.8, the compressive strengths at 7, 28, and 56 days were only 3.15, 14.00, and 16.40 MPa, respectively. Increasing the dosage by 10%, 20%, 30%, and 40% successively increased the corresponding strengths to 12.80/25.85/32.30 MPa, 23.40/41.55/48.00 MPa, 35.50/50.75/57.63 MPa, and 45.30/60.80/58.35 MPa, respectively.

Figure 8c illustrates the effect of activator modulus on compressive strength. In contrast to the monotonic trends observed for BS content and activator dosage, the activator modulus exhibited an optimum value. As the modulus increased from 0 to 2.0, the compressive strength initially increased and subsequently declined. The optimum modulus for the 7- and 28-day strengths was 0.5, corresponding to peak strengths of 35.00 and 44.05 MPa, respectively, whereas the highest 56-day compressive strength (55.35 MPa) was obtained at a modulus of 1.0. These results indicate that an appropriate activator modulus promotes the dissolution of aluminosilicate precursors and the formation of reaction products, whereas an excessively high modulus adversely affects the alkali activation process.

#### 3.2.2. XRD

**Figure 9:**
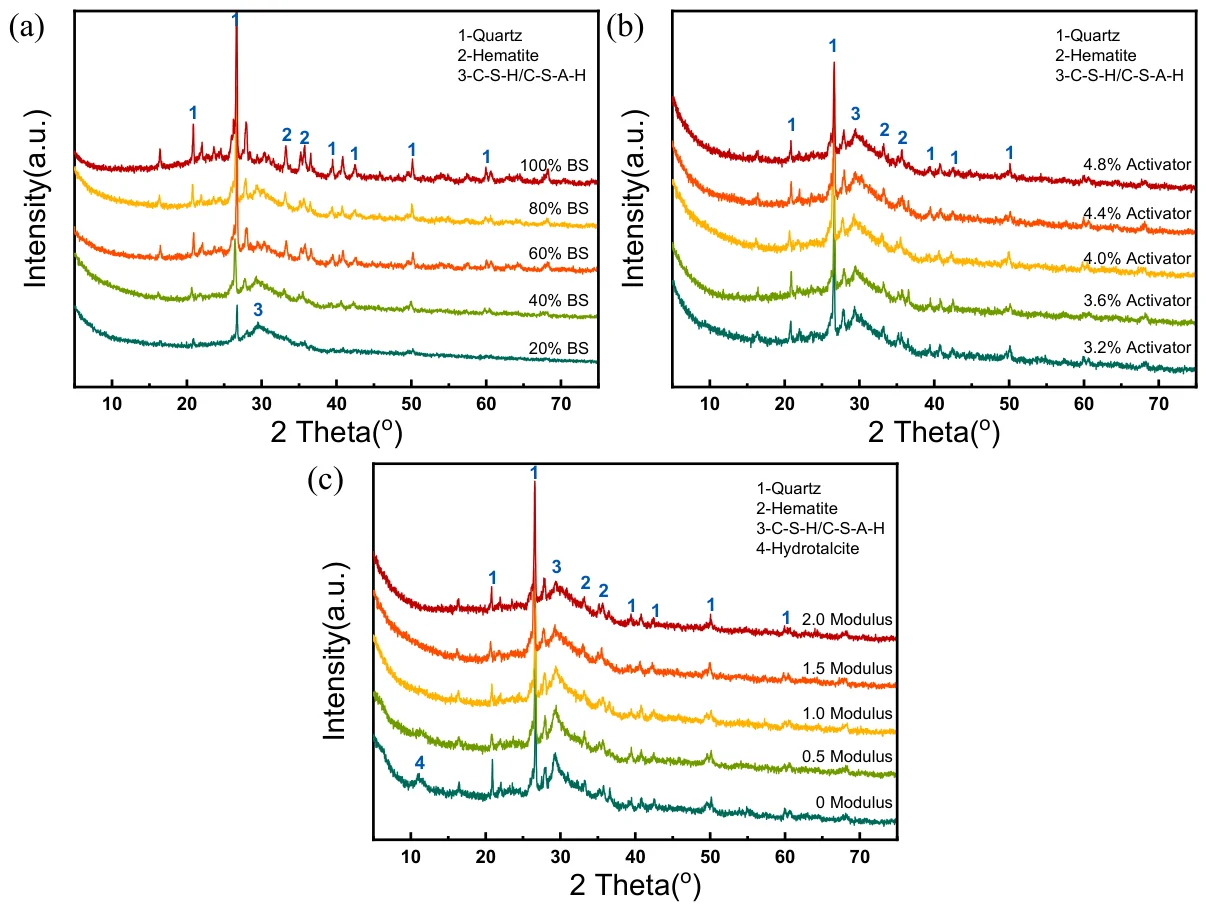
XRD patterns of BS-based AAMs. (**a**) Varying BS content. (**b**) Varying alkali activator dosages. (**c**) Varying alkali activator moduli.

Figure 9 presents the XRD patterns of the alkali-activated BS-slag binders. Quartz and hematite are identified as the dominant crystalline phases, both inherited from the BS precursor. With increasing BS incorporation, the diffraction intensity of quartz gradually increases because of the accumulation of chemically inert quartz. Since the robust Si–O framework of quartz remains essentially unreactive during alkali activation, its enrichment reduces the proportion of reactive amorphous aluminosilicate phases available for geopolymerization, thereby exerting a dilution effect on the reaction. This mineralogical characteristic is regarded as a major factor responsible for the reduction in mechanical strength at high BS contents. The broad amorphous hump centered near 29° is attributed to the coexistence of low-crystallinity hydration products, mainly C-S-H, C-A-S-H, and N-A-S-H gels [24,25].

As illustrated in Figure 9a, specimens containing 20–40% BS exhibit a broad and intense amorphous hump with a relatively large integrated area, indicating the extensive formation of amorphous gel products. In contrast, increasing the BS content to 80–100% results in a weaker and flatter hump, demonstrating that excessive BS suppresses the alkali-activation reaction and decreases the amount of hydration products formed. The evolution of the amorphous hump agrees well with the compressive strength results, showing a clear positive correlation between gel formation and mechanical performance. Consequently, the integrated area of the amorphous hump can serve as a reliable mineralogical indicator of both the hydration degree and strength development.

The XRD patterns of binders with different alkali activator dosages are presented in Figure 9b. The amorphous hump around 29° progressively intensifies as the activator dosage increases, indicating enhanced formation of amorphous hydration products. The evolution of this hump closely follows the compressive strength development, suggesting that a higher activator dosage effectively accelerates the alkali-activation reaction and promotes the generation of binding gels, thereby improving the mechanical properties of the binder.

Figure 9c compares the XRD patterns of binders prepared with different alkali activator moduli. Hydrotalcite-like diffraction peaks are observed only in systems with low activator modulus, with the strongest diffraction intensity occurring at a modulus of 0. This phenomenon can be explained by the favorable formation of hydrotalcite-like phases (approximately12°) under highly alkaline conditions [26,27]. As the activator modulus increases, the alkalinity of the reaction system decreases, thereby suppressing hydrotalcite crystallization. More importantly, the quantity of amorphous hydration products exhibits an increase followed by a decrease with increasing activator modulus, indicating that the extent of alkali activation first improves and then declines. This variation is consistent with the compressive strength results, further confirming the close relationship between hydration product formation and mechanical performance.

#### 3.2.3. SEM

**Figure 10:**
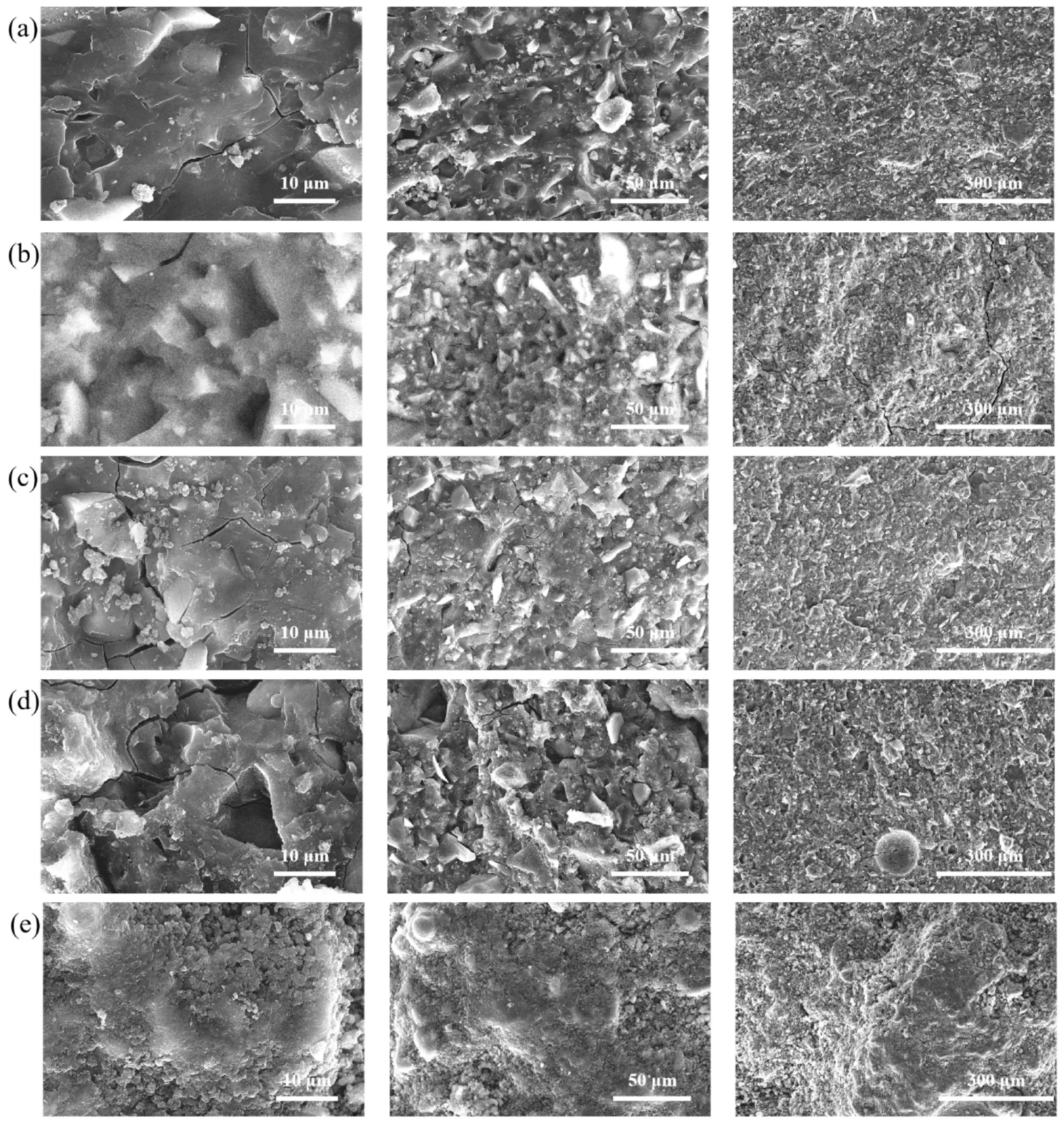
SEM of BS-based AAMs with varying BS content: (**a**) 20 wt.%, (**b**) 40 wt.%, (**c**) 60 wt.%, (**d**) 80 wt.%, (**e**) 100 wt.%.

Figure 10 illustrates the SEM morphologies of the cementitious materials containing different BS replacement levels. Progressive deterioration of the microstructure was observed with increasing BS content, characterized by increased microcrack density, enlarged pore size, and reduced matrix compactness.

At BS replacement levels of 20–40% (Figure 10a,b), the matrix exhibited a uniform and compact morphology with excellent continuity. This was primarily attributed to the high reactivity of slag, which produced abundant amorphous hydration products (C-S-H, C-A-S-H, and N-A-S-H gels). These gels effectively filled the interparticle voids and encapsulated unreacted particles, resulting in a dense microstructure. Increasing the BS content to 60% (Figure 10c) led to the appearance of additional microcracks and localized pores. The reduced formation of hydration products resulted in incomplete particle coverage, partial exposure of particle surfaces, and weakened interfacial bonding. At 80% BS replacement (Figure 10d), pronounced macropores developed, particle contacts became discontinuous, and the overall structural integrity decreased markedly. At full BS replacement (Figure 10e), the matrix contained considerable amounts of impurities and unreacted mineral phases, while only limited block-like gel agglomerates were observed. The microstructure appeared indistinct and was predominantly composed of loosely packed flocculent phases. It is noteworthy that, within the BS replacement range of 20–60%, the hydration products became progressively finer and denser as BS content increased. This evolution is attributed to the lower hydration rate and decreased Ca/Si ratio induced by BS incorporation, which promoted the formation of compact C-A-S-H and N-A-S-H gels.

**Figure 11:**
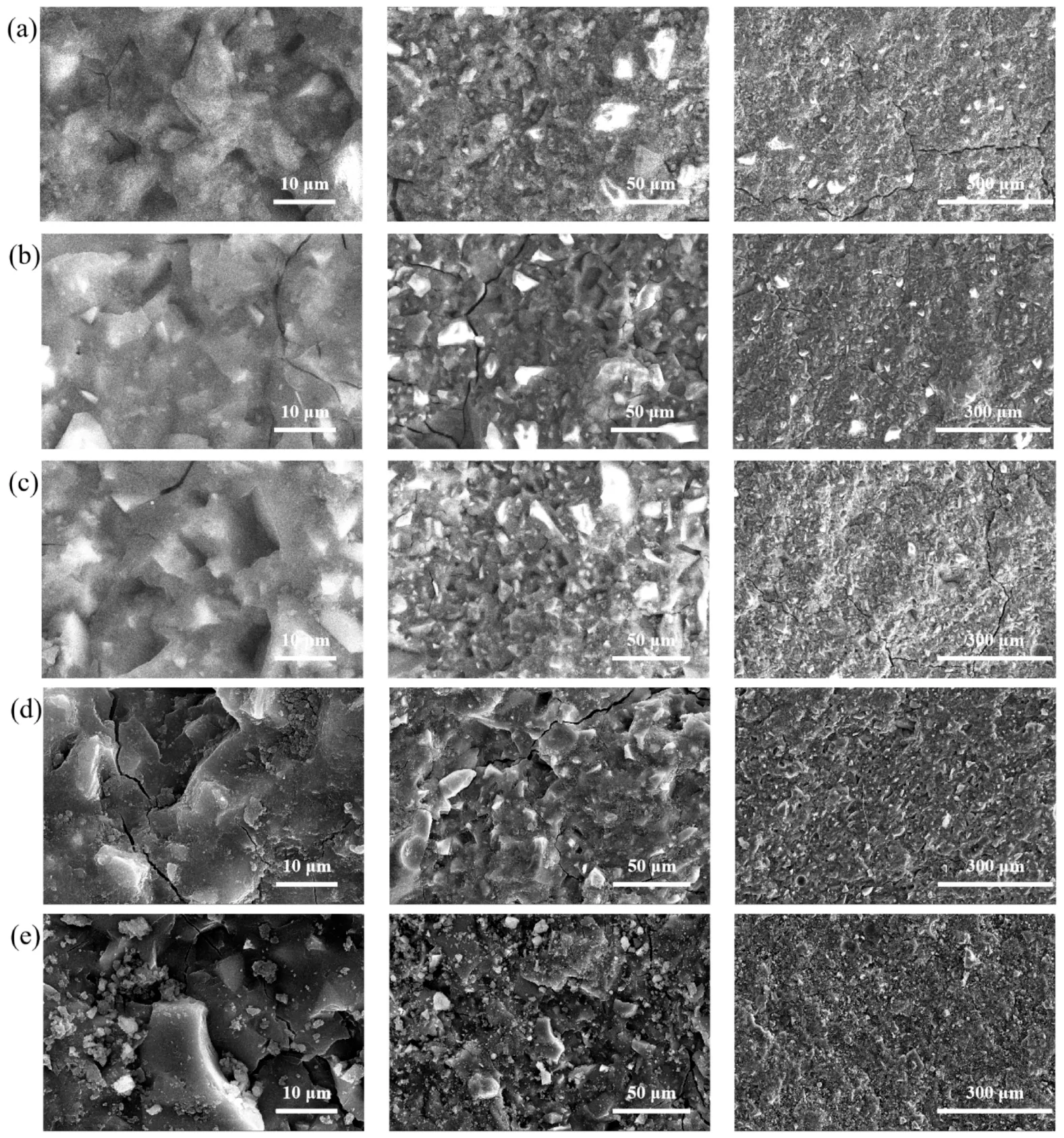
SEM of BS-based AAMs with varying alkali activator dosages: (**a**) 3.2 wt.%, (**b**) 3.6 wt.%, (**c**) 4.0 wt.%, (**d**) 4.4 wt.%, (**e**) 4.8 wt.%.

The SEM observations in Figure 11 reveal that increasing the alkali activator dosage progressively improves the compactness of the cementitious matrix while simultaneously increasing the occurrence of microcracks and enhancing the filling of interparticle pores. At an activator dosage of 3.2 wt.% (Figure 11a), the matrix is characterized by a loose morphology with poor densification and numerous interparticle voids. Increasing the dosage to 4.0 wt.% (Figure 11c) promotes the generation of interconnected bulk gel products, which gradually coalesce into continuous and smooth gel walls, producing a dense gel network with minimal microcracking and well-filled pore spaces. Further increasing the dosage to 4.8 wt.% (Figure 11e) results in localized densification of the gel phase; however, pronounced penetrating microcracks and disordered accumulation of hydration products become evident, ultimately compromising the integrity of the matrix. These microstructural changes suggest that although a higher alkali activator dosage enhances hydration and increases gel production, excessive alkalinity accelerates the reaction kinetics, leading to uncontrolled growth and random accumulation of hydration products. Consequently, structural defects are introduced into the matrix, resulting in reduced structural integrity and a decline in mechanical strength.

**Figure 12:**
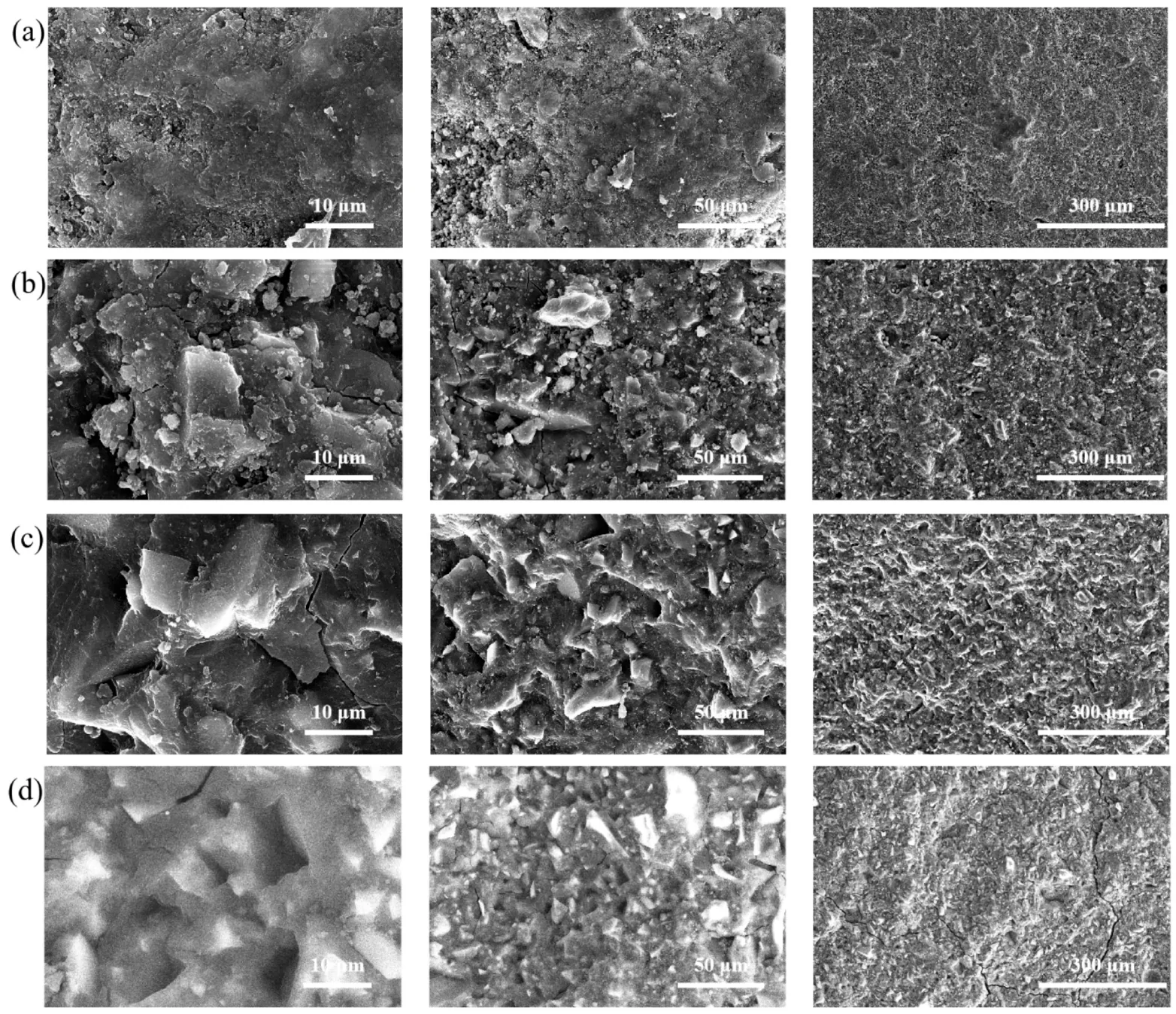
SEM of BS-based AAMs with varying alkali activator moduli: (**a**) 0 modulus, (**b**) 0.5 modulus, (**c**) 1.0 modulus, (**d**) 1.5 modulus.

Figure 12 illustrates the SEM morphologies of the cementitious materials activated with alkali activators of different moduli. The activator modulus exerted a pronounced influence on the microstructural characteristics. Increasing the activator modulus promoted the densification of the hydration matrix but simultaneously intensified microcrack development. For the specimen with an activator modulus of 0, the microstructure exhibited a porous framework composed of continuous dense regions embedded with loose and dense particles. At lower magnification, the matrix remained relatively intact and compact with few observable microcracks. The continuous dense regions were primarily attributed to the accumulation of poorly crystalline hydration products, mainly C-S-H and C-A-S-H gels, whereas the dense particles were identified as undissolved inert mineral phases inherited from the BS.

When the activator modulus reached 1.0, discrete particles were scarcely observed, and the matrix evolved into a continuous dense phase with a rough surface morphology. The overall integrity and compactness of the matrix were noticeably improved, although wider microcracks became evident. Further increasing the activator modulus to 1.5 resulted in a substantially denser hydration matrix. The surface morphology became markedly smoother, and the granular texture nearly disappeared. Nevertheless, the increased matrix compactness was accompanied by a significant increase in crack density and crack propagation.

The above microstructural evolution can be attributed to changes in the hydration chemistry induced by the activator modulus. At a modulus of 0, the limited availability of reactive silica led to a relatively high Ca/Si ratio, favoring the formation of C-S-H gel with a comparatively loose and porous morphology. As the activator modulus increased, additional reactive silica was introduced into the system, progressively decreasing the Ca/Si ratio and driving the reaction toward the formation of larger quantities of C-A-S-H and N-A-S-H gels. The accumulation of amorphous aluminosilicate gels, especially N-A-S-H gel, significantly refined the pore structure and enhanced matrix densification, thereby contributing to improved mechanical performance. However, the denser microstructure also experienced greater capillary tensile stresses during drying due to moisture evaporation, resulting in more pronounced shrinkage cracking. Such crack development partially offset the beneficial effect of matrix densification and ultimately constrained the further improvement of mechanical strength.

#### 3.2.4. Cementation Mechanism

The performance of BS-based geopolymer binders primarily depends on the alkali-induced dissolution and polycondensation of the amorphous aluminosilicate phases contained in BS and GGBFS. BS is formed by the solidification of molten coal ash at 1300–1700 °C in pulverized-coal boilers, producing irregular clinker-like particles rich in amorphous aluminosilicate glass due to the high melting temperature. GGBFS is obtained by rapid water quenching of molten blast furnace slag and generally contains more than 90% glassy phase. Under alkaline activation by sodium hydroxide or sodium silicate, hydroxyl ions break the Si–O–Si and Al–O–Si bonds within the glass network, releasing soluble silicate and aluminate species together with Ca^2+^ and Mg^2+^ into the pore solution.

These dissolved species subsequently polymerize to generate amorphous C-A-S-H and N-A-S-H gels. The C-A-S-H gel, which resembles the hydration product of Portland cement, exhibits a denser structure because of aluminum incorporation and a lower Ca/Si ratio, thereby providing the major contribution to early strength. The N-A-S-H gel is composed of interconnected SiO_4_ and AlO_4_ tetrahedra, with Na^+^ ions balancing the framework charge, resulting in a highly cross-linked and chemically stable geopolymeric network.

The coexistence of BS and GGBFS produces a significant synergistic effect during alkali activation. The high-calcium GGBFS rapidly supplies Ca^2+^, promoting C-A-S-H formation and accelerating early strength gain, whereas the low-calcium, silica/alumina-rich BS contributes additional aluminosilicate species that enhance the development of a stable three-dimensional geopolymer framework. This complementary reaction mechanism improves gel compactness and facilitates more complete utilization of the amorphous phases, yielding alkali-activated binders with superior mechanical performance and durability.

## 4. Conclusions

(1) BS is a chemically stable aluminosilicate waste with abundant amorphous phases and low carbon residue, exhibiting significant latent cementitious activity despite its relatively low early reactivity.
(2) The mechanical performance of BS-based alkali-activated binders was strongly dependent on the BS content and activator parameters. A BS content of 20–40 wt.% achieved a favorable balance between precursor reactivity and mechanical performance, while higher BS contents markedly suppressed geopolymerization and strength development.
(3) The development of compressive strength is directly correlated with the generation of amorphous C-A-S-H and N-A-S-H gels and the evolution of a dense microstructure, whereas excessive BS suppresses geopolymerization through the dilution of reactive glass phases.
(4) The synergistic activation of BS and GGBFS provides an effective strategy for converting coal-fired BS into high-value, low-carbon cementitious materials, offering substantial potential for the sustainable utilization of coal combustion solid wastes and the reduction in cement-related carbon emissions.

## Figures and Tables

**Table 1:** The chemical composition of GGBFS.

Compound	SiO_2_	Al_2_O_3_	CaO	MgO	Na_2_O	P_2_O_5_	K_2_O	TiO_2_	MnO	Fe_2_O_3_
GGBFS (%)	35.236	15.142	35.926	9.071	0.345	0.009	0.252	1.390	0.426	0.500

**Table 2:** Material ratios of alkali activator with different moduli.

Synthetic Modulus	0 Modulus	0.5 Modulus	1.0 Modulus	1.5 Modulus
Sodium Silicate (%)	0	6.40	12.80	19.20
Sodium Hydroxide (%)	10.20	8.50	6.70	4.90
Na_2_O equivalent (%)	7.982	7.974	7.964	7.955

**Table 3:** Material ratios of paste samples.

Sample	Varying BS Content	Varying Activator Modulus	Varying Activator Dosage
BS (%)	20/40/60/80/100	40	40
GGBFS (%)	80/60/40/20/0	60	60
Activator modulus	1.5	0/0.5/1.0/1.5/2.0	1.5
Activator dosage (%)	4.0	4.0	3.2/3.6/4.0/4.4/4.8
Water * (%)	35	35	35

Water * refers to the total amount of water, including that contained in the alkali activator.

**Table 4:** Material ratios of mortar samples.

Sample	Varying BS Content	Varying Activator Modulus	Varying Activator Dosage
BS (%)	20/40/60/80/100	40	40
GGBFS (%)	80/60/40/20/0	60	60
Activator modulus	1.5	0/0.5/1.0/1.5/2.0	1.5
Activator dosage (%)	4.0	4.0	3.2/3.6/4.0/4.4/4.8
Water * (%)	45	45	45
ISO sand (%)	300	300	300

Water * refers to the total amount of water, including that contained in the alkali activator.

**Table 5:** Reference compressive strength of OPC (MPa).

Curing Time	7 d		28 d		56 d	
OPC mortar	45.3	*σ* = 0.12	52.37	*σ* = 2.28	60.27	σ = 2.01

**Table 6:** Chemical compositions of BS from different power plants.

Composition	Jingtai	Jingyuan	Yuzhong	Fanping	Xigu
Na_2_O (%)	0.8786	0.7645	1.0456	0.9978	0.7610
MgO (%)	1.0969	1.3457	1.1789	1.2924	1.2323
Al_2_O_3_ (%)	21.4107	20.6548	18.3286	17.8395	19.8482
SiO_2_ (%)	57.4600	55.3538	63.4780	60.5235	59.8845
P_2_O_5_ (%)	0.2441	0.3964	0.1234	0.3529	0.3204
SO_3_ (%)	0.5798	0.6233	0.2249	0.7277	0.5503
K_2_O (%)	1.5349	1.7363	1.5596	1.9093	1.6697
CaO (%)	5.9476	6.3566	5.7783	6.0600	5.3629
TiO_2_ (%)	0.9220	0.9776	0.9167	0.9331	0.9362
Cr_2_O_3_ (%)	0.0432	0.1372	0.0592	0.1244	0.2237
MnO (%)	0.1154	0.2639	0.1010	0.1043	0.1065
Fe_2_O_3_ (%)	9.4808	10.7373	7.0825	8.8254	8.6781
NiO (%)	0.0142	0.0736	0.0203	0.0711	0.0758
CuO (%)	0.0093	0.0064	0.0070	0.0053	0.0068
ZnO (%)	0.0089	0.0072	0.0067	0.0134	0.0048
Rb_2_O (%)	0.0090	0.0085	0.0073	0.0090	0.0082
SrO (%)	0.0729	0.0652	0.0581	0.0823	0.0963
Y_2_O_3_ (%)	0.0051	0.0620	0.0034	0.0018	0.0041
ZrO_2_ (%)	0.0212	0.0292	0.0203	0.0252	0.0176
**Composition**	**Pingliang**	**Yuanyang**	**Sinopec**	**Liupanshan**	**Changle**
Na_2_O (%)	1.3518	1.1283	0.9863	0.8921	0.9560
MgO (%)	1.4740	1.6091	1.5638	1.7283	1.4045
Al_2_O_3_ (%)	24.2841	21.0738	18.0270	20.1813	23.5612
SiO_2_ (%)	54.8214	58.8152	60.9289	56.6237	53.4828
P_2_O_5_ (%)	0.4017	0.4201	0.5100	0.2381	0.1851
SO_3_ (%)	0.8857	0.7276	0.8251	1.1826	1.5436
K_2_O (%)	2.4761	2.1073	2.0090	1.8630	1.7826
CaO (%)	6.0104	5.4928	5.1729	5.7827	6.4927
TiO_2_ (%)	1.0590	1.0962	0.8973	0.8916	0.9408
Cr_2_O_3_ (%)	0.1737	0.1980	0.0917	0.1090	0.0635
MnO (%)	0.0791	0.0956	0.1635	0.2283	0.1061
Fe_2_O_3_ (%)	6.2585	7.6231	8.0373	9.9091	8.9875
NiO (%)	0.0580	0.0583	0.0623	0.0050	0.0049
CuO (%)	0.0122	0.0091	0.0109	0.0089	0.0071
ZnO (%)	0.0187	0.0098	0.0090	0.0078	0.0087
Rb_2_O (%)	0.0112	0.0103	0.0108	0.0109	0.0077
SrO (%)	0.1115	0.0793	0.1093	0.0982	0.1369
Y_2_O_3_ (%)	0.0035	0.0042	0.0051	0.0071	0.0050
ZrO_2_ (%)	0.0177	0.0152	0.0209	0.0298	0.0329

## Data Availability

The original contributions presented in this study are included in the article. Further inquiries can be directed to the corresponding authors.
